# Wear Behavior and Machining Performance of TiAlSiN-Coated Tools Obtained by dc MS and HiPIMS: A Comparative Study

**DOI:** 10.3390/ma14185122

**Published:** 2021-09-07

**Authors:** Vitor F. C. Sousa, Francisco J. G. Silva, Hernâni Lopes, Rafaela C. B. Casais, Andresa Baptista, Gustavo Pinto, Ricardo Alexandre

**Affiliations:** 1 ISEP—School of Engineering, Polytechnic of Porto, Rua Dr. António Bernardino de Almeida, 431, 4200-072 Porto, Portugal; vcris@isep.ipp.pt (V.F.C.S.); hml@isep.ipp.pt (H.L.); rbc@isep.ipp.pt (R.C.B.C.); absa@isep.ipp.pt (A.B.); gflp@isep.ipp.pt (G.P.); 2 INEGI—Instituto de Ciência e Inovação em Engenharia Mecânica e Engenharia Industrial, Rua Dr. Roberto Frias, 400, 4200-265 Porto, Portugal; 3 TEandM—Technology, Engineering and Materials, S.A., Taveiro Industrial Park, 3045-508 Taveiro, Portugal; ricardo@teandm.pt

**Keywords:** duplex stainless-steel, milling, TiAlSiN, dc MS, HiPIMS, surface roughness, wear mechanisms, wear performance, adhesion, abrasion

## Abstract

Duplex stainless steels are being used on applications that require high corrosion resistance and excellent mechanical properties, such as the naval and oil-gas exploration industry. The components employed in these industries are usually obtained by machining; however, these alloys have low machinability when compared to conventional stainless steels, usually requiring the employment of tool coatings. In the present work, a comparative study of TiAlSiN coating performance obtained by these two techniques in the milling of duplex stainless-steel alloy LDX 2101 was carried out. These coatings were obtained by the conventional direct current magnetron sputtering (dc MS) and the novel high power impulse magnetron sputtering (HiPIMS). The coatings were analyzed and characterized, determining mechanical properties for both coatings, registering slightly higher mechanical properties for the HiPIMS-obtained coating. Machining tests were performed with varying cutting length and feed-rate, while maintaining constant values for axial and radial depth of cut and cutting speed. The surface roughness of the material after machining was assessed, as well as the wear sustained by each of the tool types, identifying the wear mechanisms and behavior of these tools, as well as registering the flank wear values presented for each of the tested tools. The HiPIMS-obtained coating exhibited a very similar behavior when compared to the other, producing similar surface roughness quality. However, the HiPIMS coating exhibited less wear for higher cutting lengths, proving to be a better choice in this case, especially regarding tool life.

## 1. Introduction

By combining both the benefits of having α- ferrite and γ-austenite phases, Duplex Stainless-Steels (DSS) present excellent mechanical and corrosion resistance properties, making them a very appealing material [1]. They have seen wide application in industries that benefit from these excellent properties, having various applications for a broad variety of industries [2,3]. DSSs exhibit better overall properties than more common stainless steels, resulting in a growing preference for the use of these alloys over other stainless steels, and even some nickel-based alloys [4]. DSS alloys are known to be hard-to-machine materials being characterized by having a low machinability (when compared to other materials) [5]. As such, it is advised the use of coated tools with the right geometry. Additionally, the use of coolant is advised when machining DSS alloys. The cutting forces reached during machining operations of DSS tend to be high, promoting rapid tool wear and causing damage to the workpiece surface integrity [6]. The machining process is still widely used in the production of high-precision parts, thus, the machining optimization of these alloys is quite appealing, as it may lead to better ways to produce higher quality parts at a lower cost. As seen in [6], there are some authors that propose a set of methods to machine DSS alloys. However, these methods are usually devised based on empirical data, obtained by analyzing a vast number of practical studies, which can be costly. However, there are some studies that propose a different optimization approach, with techniques such as the Taguchi method, which is commonly employed in the optimization of machining parameters [7,8,9,10]. Zhang et al. [11] used the Taguchi method for the surface roughness optimization in an end-milling operation. The authors defined the control factors as being depth of cut, feed rate, and spindle speed, concluding that the latter was the most influential parameter on the machined surface finish quality. Additionally, tool wear was found to be a significant factor on the machined surface roughness value. Selvaraj [12] also used the Taguchi method to identify the various machining parameters that influenced the cutting forces generated when dry end-milling a cast DSS alloy. It was concluded that feed rate was the most impactful parameter, having an impact of 46% in the generated cutting forces. Multiple regression analysis can also be used to obtain information regarding machining parameter influence. Airao et al. [13] evaluated the surface roughness of a Super Duplex 2507 stainless steel in dry and wet milling conditions. After the milling tests, it was concluded that the most influential parameter on the surface roughness was feed rate, followed by cutting speed. Furthermore, the optimal cutting parameters for the best machined surface roughness were defined, highlighting that, for this material, the lowest surface roughness value was obtained for high cutting speeds and low feed rates. This conclusion was also reached by Policena et al. [14], where the authors perform end-milling finishing operations on DSS alloy, UNS S32205.

The machining industry’s focus is still process optimization, as it leads to a lower cost production, thus, this is still a very popular research topic. Although the optimization of machining parameters is of high importance, the employed tool is of equal importance, as it directly impacts process efficiency. Most machining tools employed in the industry and under study are coated tools or coated carbide inserts [15,16]. Tool coatings have many benefits for machining applications, such as, improved surface finish, tool-life, and overall process stability, such as cutting force reduction [17]. These coatings improve the wear behavior of the coated tools by lowering the coefficient of friction [18], greatly improving the wear resistance of coated tools and substrates [19,20], enabling these coatings to be used in a wide variety of applications that are subject to intense wear, such as in injection molds [21,22,23]. Tool coatings are usually obtained by two main deposition processes: Physical Vapor Deposition (PVD) or Chemical Vapor Deposition (CVD). CVD films are obtained by having a precursor pumped inside a reactor. Its molecules pass by the substrate and are deposited on its surface, thus creating a CVD film, usually with a uniform thickness throughout the surface [24]. This process runs at temperatures of up to 900 °C, which makes it less suited for coating deposition on steel substrates; however, this is possible, with some diamond coatings being successfully applied to carbide and nitride substrates [25,26]. For PVD, the process runs at about 500 °C, additionally, this process is more environmentally friendly, due to the use of nontoxic materials and having a lower energy consumption when compared to CVD [27]. There is quite a variety of studies made on the comparison of coatings obtained by these two deposition methods. These studies focus on the advantages of using a certain deposition method for a certain application, such as using PVD coatings for finishing operations over CVD coatings [15,16], as concluded by Ginting et al. [28], when studying the machining of AISI 4340. The used PVD coatings usually lead to a better-quality surface finish, when compared to multilayered CVD coating. However, there are some exceptions, especially for Ni-based alloys. Koseki et al. [29], studied this machining case, concluding that due to the Ni-based super alloy’s properties, a coating able to support higher cutting forces and cutting temperatures would be more suited for the test, concluding that a CVD multilayered coating would be ideal for this case. Coating’s mechanical properties, structure, and microstructure greatly influence the machining process, directly affecting its performance [30]. Chemical composition also influences the quality of the machining process, as seen in the study by Paiva et al. [31], where a comparison of the wear performance of PVD, TiCN and TiAlN, coatings and CVD, TiCN/Al_2_O_3_, when machining a superduplex stainless steel alloy is performed. The authors concluded that the coating that exhibited less wear was the AlTiN PVD coating. This was due to the presence of Al in the coating, which significantly increased the coating’s wear behavior. This was because the presence of this element enabled for an alumina tribo-ceramic film to form during machining, causing a significant reduction in friction. The deposition method has a great influence as well, as PVD usually produces thinner films, when compared to CVD. This fact, coupled with the residual compressive stresses that are characteristic of the PVD process, confers the PVD-coated tool with a high strength coating, with very sharp cutting edges, making it usually more suited for finishing operations [32,33], with many coatings for machining applications being obtained by this process [15,16]. The most used deposition process for coatings is the direct current magnetron sputtering (dc MS); however, there is a relatively new PVD technique that seems highly promising, high power impulse magnetron sputtering (HiPIMS), particularly due to its higher ionization rates and higher charge states of the target ions [34]. This technique exhibits high potential to produce coated machining tools, enabling a higher control of deposition parameters [35], unlocking the capability to produce tailored coatings with excellent mechanical properties [36]. Coatings obtained by this PVD technique exhibit higher values of hardness, when compared to direct current magnetron sputtering [37]. Furthermore, there is also an improvement in coating adhesion to the substrate when using this technique, as well as an increase in residual compressive stresses [37,38].

The cutting forces that are generated during the machining process provide important information regarding the overall state of the process. This means that analyzing cutting force data can provide a way to monitor the machining process or even identify certain aspects of it, which can be improved. The knowledge of these cutting forces can be used to monitor tool behavior [39,40] and to provide information on optimal machining parameters [41,42], because selecting these parameters the machining process is improved. There are two main methods of determining cutting forces, using a direct or indirect approach; however, the most employed method is a direct one using a dynamometer to measure these forces. There are also developments of new predictive methods or models used for the prediction of these cutting forces, especially using Finite Element Method [43], providing a cheaper alternative for cutting forces prediction and tool behavior.

The analysis of the wear mechanisms sustained by cutting tools brings many advantages, enabling the optimization of the machining process, by improving tool-life, creating new tool geometries, and even optimizing the coatings of these tools by providing information on the right coating to use for a certain application [44]. There are studies made in this regard, focused on DSS, with common wear mechanisms being registered, such as abrasion and adhesive wear, this is due to the high strength of the material and high friction values reached during machining of these alloys [45,46,47]. Studies such as these are highly important for a proper process optimization, as knowledge about tool wear behavior enables for a better understanding on what is occurring during machining, highlighting the correct path for an improved process, either by the implementation of a new cooling method, adjusting machining parameters and selecting of the right tool for the job (prioritizing tool-life, surface roughness, and overall process efficiency), considering tool geometry and coating [45,48,49,50].

The good performance of TiAlN-based coatings for high-speed machining applications is well documented, producing good machined surface quality and increasing the tool’s wear resistance [51]. In particular, TiAlSiN coating exhibits good wear behavior and performance when used in machining operations of hard-to-machine materials, such as DSS or even hard tool-steel [52]. There is also some documentation regarding the increase in coating’s mechanical properties for coatings deposited by the HiPIMS technique when compared to the conventional dc MS technique [53]; however, there is still a low amount of research made on the testing of these coatings in the machining of DSS alloys. Furthermore, since the HiPIMS technique is highly versatile, enabling for higher deposition parameter control, studies performed in this area can improve the knowledge of the machining of these hard-to-machine materials, including DSS alloys. To further increase the knowledge of the performance of these coatings, a comparative study on the wear behavior and machining performance of TiAlSiN-coated tools was conducted, based on the previously conducted study [52], and expanding on the knowledge already acquired from that work. The coatings were obtained by two PVD techniques, direct current magnetron sputtering (as described in [52]) and high-power impulse magnetron sputtering, being applied to solid carbide end-mills. These tools were then tested in the machining of DSS alloy, LDX 2101. The machined material’s surface was evaluated, and the tool’s wear was assessed. With the results obtained in this work, the authors hope to help fill the gap regarding the research made about the machining of these type of alloys, offering a comparison of PVD techniques and their influence on the coating’s performance, contributing with information that could be relevant when it comes to the optimization of the machining process of DSS alloys.

## 2. Materials and Methods

The different materials and methods used during this study are going to be presented in the following subsections, divided and organized in a clear manner, to provide a good understanding of each of the used materials and techniques.

### 2.1. Materials

#### 2.1.1. Machining Tools

Two tool types were used for the machining tests carried out in this work. The tools substrate is cemented carbide WC-Co grade 6110, provided by INOVATOOLS, S.A. (Leiria, Portugal). The cemented carbide presents a grain with a dimension of about 0.3 µm and uses 6% Co (wt.) as a binder. The employed tools had the same geometry and dimensions, having 4 mm in diameter and a total length of 68 mm. Each tool was coated with TiAlSiN; however, the deposition process was different for each tool type, one being coated by the conventional dcMS PVD technique and the other deposited by the HiPIMS technique. Both tools presented the same geometry, being end-mills with a 0.2 mm corner radius, with four flute and a rake and relief angle of 35° and 10°, respectively. To differentiate between the two tools, the tool coated with the TiAlSiN obtained by dcMS was denominated T1, while the tool coated by the HiPIMS technique was denominated T2. After tool production and before the coating process, these were subjected to an ultrasonic bath using acetone for 10 min, followed by a second one, lasting 5 min, while changing the cleaning agent between baths.

Regarding coating deposition, a CemeCom CC800 /9ML PVD Magnetron Sputtering equipment, for the deposition of the conventional TiAlSiN coating (T1 tools), while a CemeCom CC800/HiPIMS was used to obtain the T2 TiAlSiN-coated tools. The deposition parameters for both dcMS and HiPIMS TiAlSiN coatings can be seen in the following Table 1.

The deposition parameters were selected based on previous successful experiences carried out on similar substrate materials. After the deposition process, all tools were carefully packed, avoiding direct handling of the cutting area.

#### 2.1.2. Machined Material

The machined material is a Duplex Stainless Steel, LDX 2101, which was provided as a round bar, having 80 mm in diameter. Regarding this material’s mechanical properties, it presents as yield strength 450 MPa and ultimate tensile strength 650 MPa, following the information provided by the material’s manufacturer (Outukumpu). This steel has an average hardness of 280 ± 20 HV_5_, and its chemical composition was provided by the manufacturer and can be observed in Table 2. The hardness values were confirmed by performing ten measurements in a universal hardness testing equipment, EMCO M4U, using a 5 kgf and a dwell time of 30 s. The hardness values were in accordance with those present in the material’s data sheet (290 HV_5_), exhibiting just a slight deviation.

### 2.2. Methods

#### 2.2.1. Sample Preparation

To characterize both coatings, samples were prepared, which were obtained from cutting each of these tools, enabling the analysis of their cross-section though scanning electron microscopy (SEM). The cut was performed using a Struers disc saw with embedded electroplated diamond particles along its edge. Cut samples were then embedded in thermoset resin and hot pressed in a Struers Pedopress equipment, and then subjected to sanding and polishing operations. Grinding was performed using sandpapers of decreasing granulometry, doing the following sequence: 220, 500, 800, and 1200 grit. After the use of each sandpaper reference, the samples were rotated 90 degrees, improving the reduction of surface grooves caused by the previous operation. To further improve the samples’ surface morphology, two sequences of polishing were performed using diamond slurries of 3 and 1 µm (in this order), during approximately 15 min for each polishing sequence.

#### 2.2.2. Coating Thickness Evaluation

Tool coating thickness evaluation and measurement was performed on the prepared samples, using a FEI QUANTA 400 FEG scanning electron microscope (SEM), provided with an EDAX Genesys Energy Dispersive X-Ray Spectroscopy microanalysis system. The EDS analysis was carried out using a beam potential of 15 kV; however, this was sporadically reduced to 10 kV to reduce the amount of noise in the spectra by reducing the volume of interaction. In terms of quantitative analysis, the accuracy of the EDS analysis is not the best; however, it was considered sufficient in its use for the confirmation of the coating’s chemical composition, thus avoiding the employment of a more costly technique.

#### 2.2.3. Machining Tests

##### Machining Strategy

The machining tests were conducted in a milling center, HAAS VF2 CNC machine, having a maximum power of 20 kW and capable of reaching 10,000 rpm. Given the raw material geometry, a strategy in which the tool would machine the material’s surface in a spiral motion was adopted, moving from the periphery of the material to its center. The machining tests were performed using a water-miscible cutting fluid projected externally (5% oil in water). The machining parameters were determined based on the suggestions of the tool’s substrate provider, being applied to both tools, thus enabling the comparison of their performance and wear behavior. These parameters are presented in Table 3. A total of three tests for each cutting condition were performed, producing a greater number of results, thus improving the results’ consistency.

Regarding the machining parameters, a constant value for radial depth of cut and vertical depth of cut was used, this being 3 and 0.08 mm, respectively. Moreover, a constant cutting speed of 60 m/min was used. To evaluate the influence of feed rate value on the machined surface roughness and on the tools’ wear, this parameter was varied by 25%, creating test conditions that employed 75% and 125% of the originally recommended feed rate value, this being 479 mm/min. To determine the optimal cutting length value to carry out this comparative experimental work, cutting force analysis was used. For this purpose, one of each produced tool was used in the machining of the material, using the strategy and recommended parameters described above. For this preliminary test, a cutting length of 25 m was adopted, being this procedure described in detail (mentioning the equipment) in the following subsection.

##### Cutting Force Analysis

Valuable information regarding the tool’s wear and cutting performance can be obtained by analyzing the cutting forces that are developed during the machining process. To acquire these forces, a four-component KISTLER 9171A dynamometer was used, coupled to a KISTLER 5697A1 data acquisition system. This allowed the recording of the cutting forces developed in X, Y, and Z axes, as well as the developed torque (Mz). These values are obtained and interpreted through appropriate software, supplied with the dynamometer. The equipment was coupled to the spindle of the CNC milling center, having then the appropriate clamping system for placing the tool. The acquisition rate of the cutting forces was selected according to the spindle’s rotational speed, enabling to register the force value for all the tool’s cutting edges at each rotation. The methodology for cutting force acquisition described in [52] was adopted as well for this work, enabling once again to detect an abnormal behavior of these cutting forces, realizing that after 4 m of cutting length, the tools presented high levels of wear. Under some conditions, there were wear phenomena noticeable even after 2 m of cutting length. Thus, two additional cutting conditions were created, one for 2 m of cutting length and another for 4 m.

##### Machining Test Parameters

With the cutting lengths determined, the main wear mechanisms that these tools are subject to could be identified and evaluated. The defined machining parameters for both tool types can be observed in Table 3, the values for cutting speed, depth of cut, and width of cut were kept constant, these being 60 m/min, 0.08 mm, and 3 mm, respectively.

Three tools were used for each of the conditions presented in Table 3. After machining, all the tools underwent an ultrasonic bath using acetone, for 5 min, to remove the presence of lubricant used during the tests. This bath had a short duration to prevent the eventual removal of adhered material or sections of coating (near detachment). The surface roughness of the material was also assessed after each machining test.

#### 2.2.4. Surface Roughness Evaluation

The surface roughness of a machined material can provide valuable information regarding machining performance, tool wear, and overall process stability. Surface roughness evaluation was performed on the material’s machined surface, evaluating the value of this parameter in two directions: radial and tangential (to the machining direction). The surface roughness tests were conducted using a MAHR PERTHOMETER M2 profilometer, following the standard procedure described in DIN EN ISO 4288/ASME b461. Each test covers a length of 5.6 mm, corresponding to seven segments of the cut-off value (0.8 mm), with the first and last value being ignored due to the acceleration and deceleration of the probe arm. The considered parameters for the surface roughness analysis were arithmetic mean roughness (Ra) and the maximum roughness (Rmax). Furthermore, the R profile was analyzed, to identify any sharp peak or strange phenomena on the surface roughness values.

#### 2.2.5. Tool Wear Analysis

The tools that were used in the milling tests were subjected to SEM analyses to access the amount of sustained wear and identify the main wear mechanisms that developed during machining. To quantify the wear that these tools sustained, the values for flank wear were measured (VB), following the ISO 8688-2:1986 standard [54], which will be presented for each tool and tested condition.

**Figure 1:**
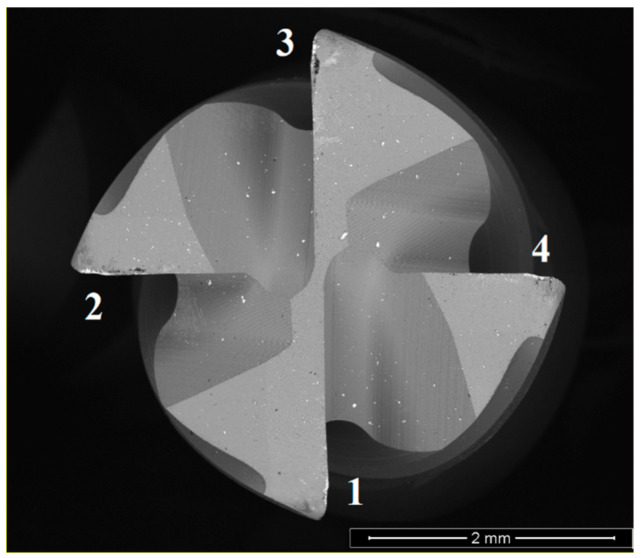
Reference for SEM analysis of the coated tools.

The reference used for tool analysis can be observed in Figure 1. Both the rake and clearance face were analyzed, identifying the main wear mechanisms, and measuring the flank wear (VB) in the tested tools’ clearance faces.

The numbers depicted in Figure 1 were adopted in the identification of the SEM images, with the rake and clearance faces being identified with RF and CF, respectively, indicating the edge under analysis as presented in Figure 1. Since three tools were used to characterize each test condition, an identification number, ranging from 01 to 04, was added at the end of each image reference.

## 3. Results and Discussion

In the current section, the obtained results from each of the analyses described in the previous section are going to be presented. Each of the main results are divided into different sections, from the characterization of both TiAlSiN coatings, to the analysis of the registered tool wear mechanisms.

### 3.1. Coating Characterization

The thickness of both TiAlSiN coatings were analyzed using the methodology described previously. Coating thickness values were obtained by calculating the mean value of all the performed measurements. Six measurements were performed across different zones of the sample. The average thickness of each of the analyzed coatings can be observed in Table 4. Coating mechanical properties were also evaluated, namely hardness and Young’s Modulus. These values were obtained by performing ultra-micro hardness tests on both TiAlSiN coatings, using a dynamic ultra-micro hardness tester, Fischerscope H100. The samples used for this test were obtained by placing flat substrate samples together with the uncoated substrate tools, during the deposition process. Regarding the parameters used for this test, a normal maximum load of 50 mN was used, with dwell time of 30 s. A total of ten tests per sample were performed, enabling for the calculation of the average values of hardness and Young’s Modulus for each coating, as seen in Table 4.

### 3.2. Cutting Force Analysis

Cutting forces were registered for all the tested cutting conditions; however, due to the values of the chosen machining parameters, primarily due to the low depth of cut, the registered cutting forces did not present high values.

Analyzing all the cutting force graphs, a common trend could be identified. Regardless of feed rate, the cutting forces would increase in value for the Fx, Fy, and Fz components, with the latter increasing more significantly throughout the machining tests. As registered in the preliminary tests, this cutting force increase could be seen at around the 2-m cutting length mark for the tested tools. Regarding the Mz force component, the registered values were very small, and its behavior remained constant throughout the conducted tests.

### 3.3. Surface Roughness Analysis

In this subsection, the results regarding the machined material’s surface roughness are going to be presented. To evaluate machining stability, both the values obtained for radial and tangential direction are going to be presented, as a large difference between these values usually indicates excessive tool vibration during the machining process.

Regarding the obtained surface roughness values for tests carried out using T1 (dcMS TiAlSiN-coated tool), these can be seen in Table 5.

Analyzing the values shown in Table 5, it can be concluded that the machining process is quite stable, as there is no significant deviation in surface roughness value measured in both directions. There is an increase in surface roughness for higher values of cutting length, with the 2-m cutting length condition producing the best results in terms of surface roughness for the different tested feed rate values. Regarding the influence of feed rate in these values, it seems that an increase in feed rate causes a deterioration in surface quality. The lowest value of surface roughness value was registered for the lowest feed rate value, for 2 m of cutting length.

Regarding the values obtained for the tests conducted with T2 (HiPIMS TiAlSiN coated tools), these can be observed in Table 6.

From Table 6, the surface roughness values are quite like those obtained using T1. The process is stable, showing no major difference in radial and tangential surface roughness values. As seen for tests conducted using T1, the best surface quality is obtained for lower cutting length and feed rate values, namely 2-m cutting length and 75% of feed rate.

#### Results Comparison

**Figure 2:**
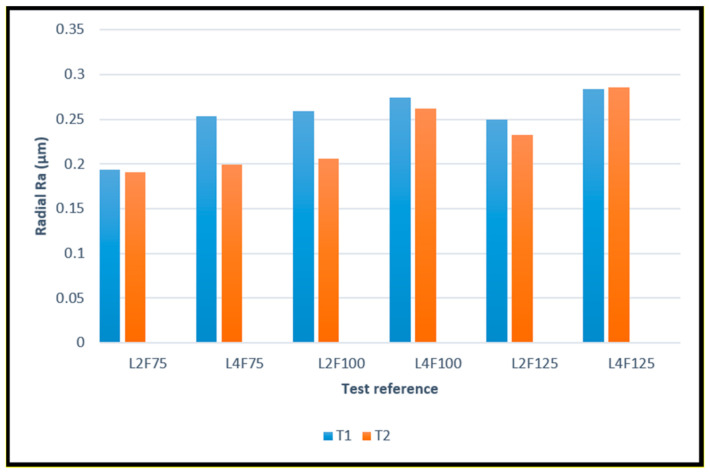
Average values of Ra, measured in the radial direction, produced by T1 and T2 tools for all the different test conditions.

**Figure 3:**
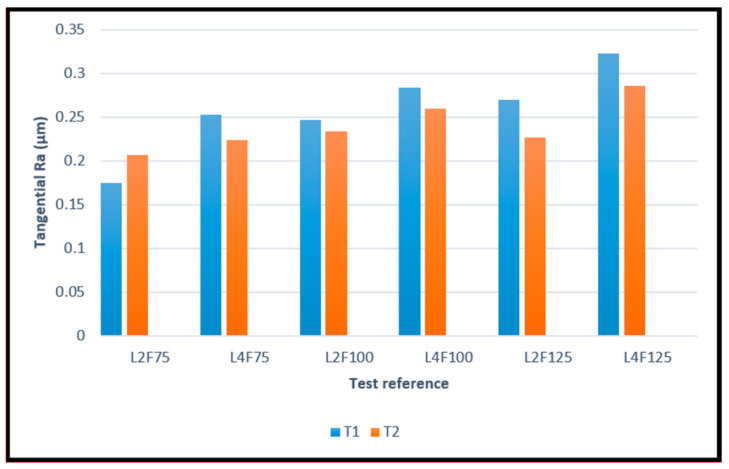
Average values of Ra, measured in the tangential direction, produced by T1 and T2 tools for all the different test conditions.

The surface roughness values obtained for both directions and coated tools can be observed in Figure 2 and Figure 3, as bar graphs, enabling their comparison. The images depict the values for all tested conditions, with the number after “L” indicating the cutting length used, and “F” indicating the percentage of feed rate used during the machining test.

The obtained values for both coated tool types were very similar, with the T2-coated tools slightly outperforming the T1-coated tools, producing an overall better surface roughness. It is worthy to note that these values are quite satisfactory. Despite having different values, the surface roughness produced by both tool types behaved in the same manner, increasing with cutting length and feed rate. This is to be expected, as the longer the tool operates, the more wear it sustains, resulting in a worse surface quality. Furthermore, feed rate is a very impactful parameter on the machined surface quality [13,14]. It is also worth noting that the surface quality produced by T2 tools deteriorated less significantly over the cutting length, when compared to the T1 tools.

The better performance by the T2 tools can also be attributed to the coating’s residual compressive stresses, as HiPIMS usually produces coatings with higher values of these stresses, when compared to PVD dc MS deposition technique [37,38]. Moreover, these stresses can be beneficial to produce a better machined surface quality [51].

### 3.4. Tool Wear Evaluation

In this subsection, the wear analysis of both tool types is going to be presented. The SEM images of the tested tools were analyzed, performing flank wear (VB) measurements on the tool’s clearance faced. Furthermore, the main wear mechanisms that were present were identified. Firstly, the wear measurements for both T1 and T2 tools are going to be presented, followed by a section devoted to the analysis of the wear mechanisms sustained by the tools. Finally, there will be a section that will offer a comparison of the measured wear values for each of the tool types, while commenting on the registered information.

#### 3.4.1. Wear Measurements

Regarding the average values of flank wear measured in T1 tools for all tested conditions, these values can be observed in Table 7.

From Table 8, the flank wear increases with cutting length, especially at 75% and 100% feed rate values. In terms of flank wear variation with feed rate, there is an increase in wear from 75% to 100% feed rate value; however, at higher feed rate values the sustained wear is minimum. It is worth noting that the amount of sustained wear by these tools is quite low, given the machined material, highlighting the high wear resistance of the TiAlSiN coating.

The values of flank wear measured for the T2 tested tools can be observed in Table 8.

As seen for the values of machined surface roughness, the measured T2 flank wear values follow a similar trend to those registered for T1 tools. There is an increase in flank wear for higher cutting lengths, furthermore, the wear is minimum for higher values of feed rate, as seen for T1 tools. However, in the case of T2 tools, there seems to be a lower variation in flank wear for higher values of cutting length. For T2, the testing condition that produced less flank wear was the same as T1, 2 m cutting length at 125% feed rate value.

#### 3.4.2. Tool Wear Mechanism Analysis

**Figure 4:**
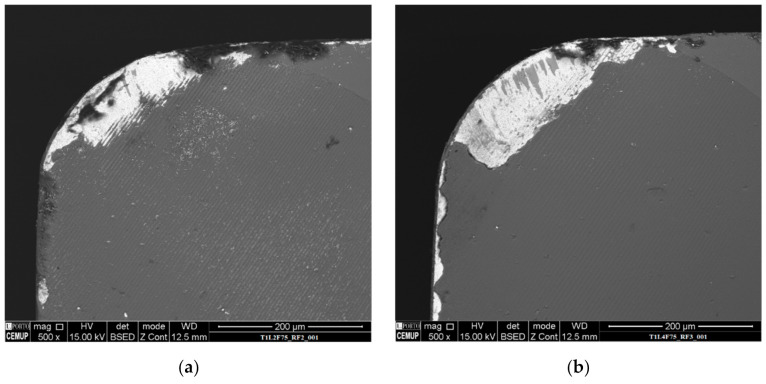
Rake face, with a 500× magnification, of the (**a**) T1L2F75; (**b**) T1L4F75 tested tools.

Both coated tools exhibited the same wear behavior, as seen from the measured flank wear values. The wear in the flank increased with the cutting length for all the tested feed rate conditions, and this was also registered in the rake face, as seen in Figure 4, where the rake face wear for T1 tools tested at 2- and 4-m cutting length at 75% feed rate can be observed.

This wear behavior was registered for all the tested conditions for both T1 and T2 tool types, albeit in different intensities, with all tools tested at higher cutting lengths exhibiting higher rake face wear. Furthermore, it was registered that the wear behavior of the rake face was the same as the flank wear behavior.

The main wear mechanisms registered were adhesion, abrasion, and coating delamination, for both T1 and T2 tools. However, the intensity of these mechanisms was not as intense in the T2-coated tools, with the T1 tools exhibiting much more developed stages of wear.

**Figure 5:**
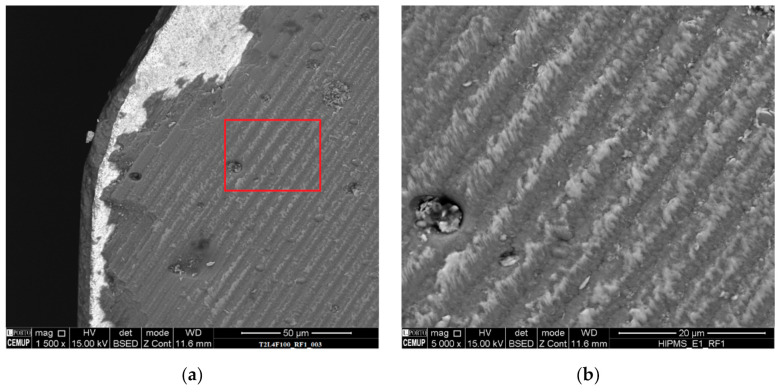
Rake face, with a 1500× magnification, of the (**a**) T2L4F100 tool; (**b**) magnification of the highlighted area.

Due to the material’s properties, it tends to adhere to the tool’s surface during machining, as can be seen in Figure 5, which depicts adhered material on a T2 tool’s rake face.

**Figure 6:**
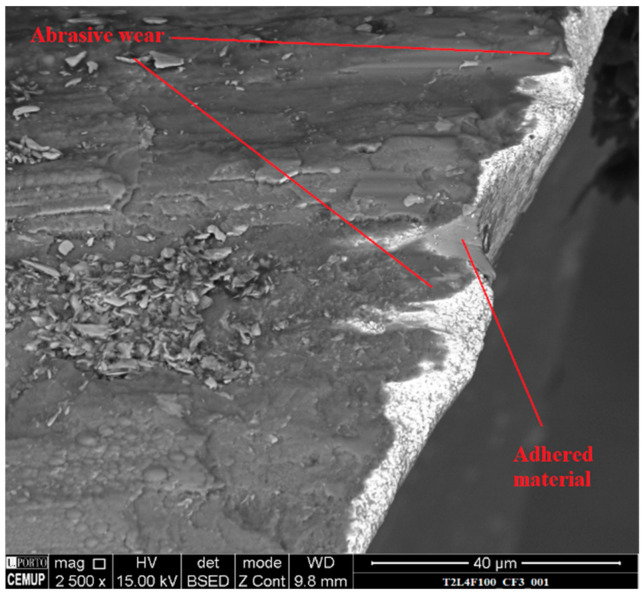
SEM image of the clearance face of T2L4F100 tool at 2500× magnification.

**Figure 7:**
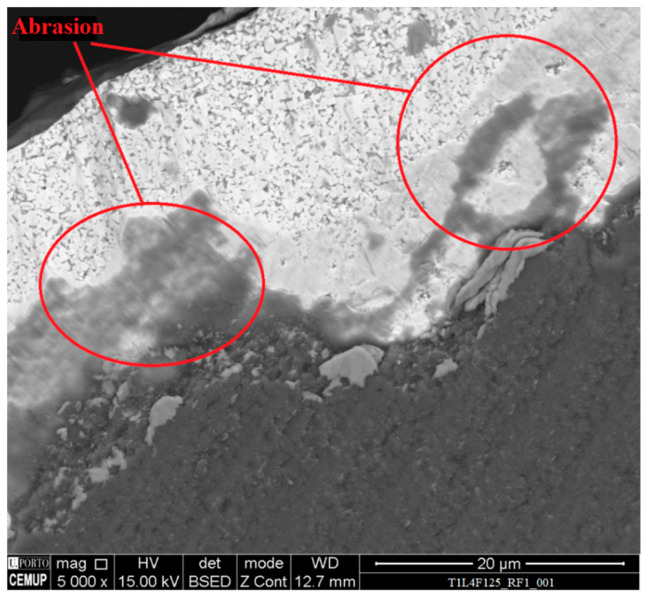
SEM image of the rake face of a T1L4F125 tested tool, at 5000× magnification.

Abrasion was also registered on all the tested tools; however, this mechanism was more apparent for higher values of cutting length. Figure 6 and Figure 7 depict abrasive wear on a clearance and rake face of analyzed tools. Although abrasive wear was detected in T2 tools, in this case the wear damage was less intense than that registered for the T1 tools.

**Figure 8:**
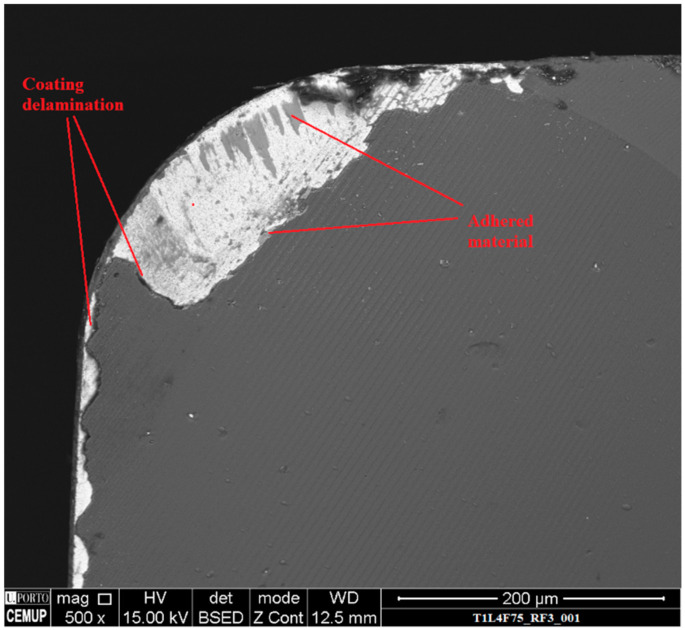
Rake face, with a 500× magnification, of the T1L4F75 tested tool, with wear mechanism identification.

The third, and last main wear mechanism registered was coating delamination, eventually resulting in substrate exposure. This can be observed in Figure 8. Coating delamination was observed in all the tested tools, with tools tested at higher values of feed rate and cutting length exhibiting more delamination damage.

**Figure 9:**
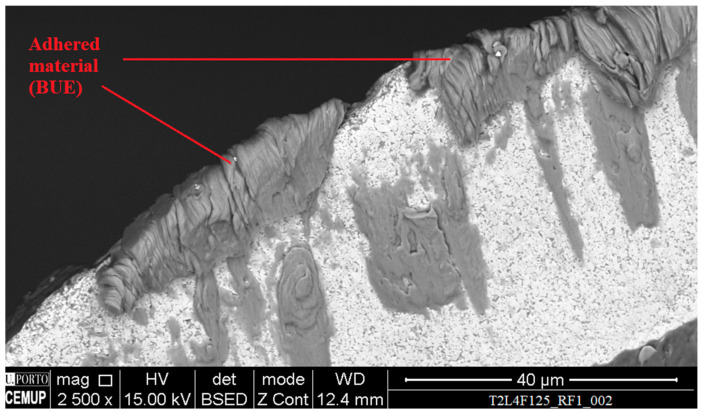
Rake face, with a 2500× magnification, of the T2L4F125 tested tool, with wear mechanism identification.

Some cases of built-up edge were registered, caused by a high amount of adhered material to the cutting edge, an example of this phenomenon can be observed in Figure 9. However, this was not persistent for all the tools, presenting itself primarily for higher values of feed rate.

**Figure 10:**
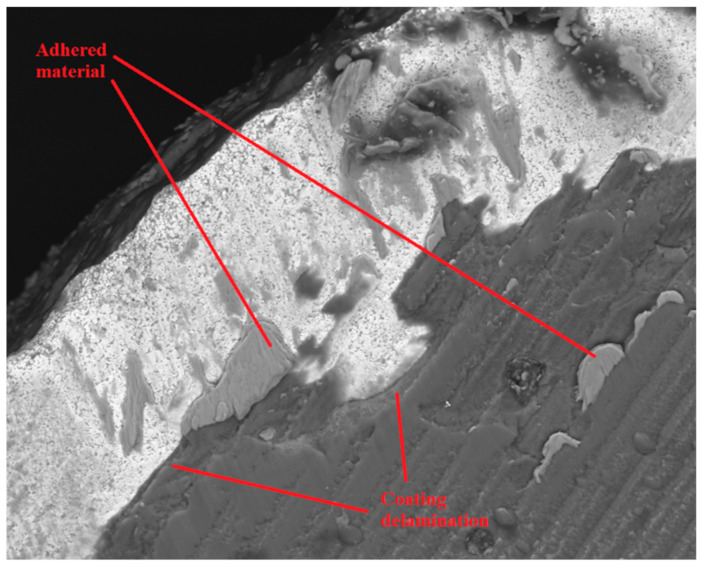
Material adhesion to delaminated areas of the TiAlSiN coatings, rake face of a T2L4F100 tool at 2500× magnification.

Regarding the evolution of the registered wear mechanisms, it was concluded that the adhesive wear is the wear mechanism that is firstly developed. The adhered material in the coating’s surface promotes abrasive wear in the area, and this abrasive wear induces coating delamination. After coating delamination, the material tends to adhere to the border between the delamination and the tool’s substrate, as can be seen in Figure 10.

This adhered material will promote more abrasive wear in this border area, causing further coating delamination, eventually leading to total coating failure. This was also registered in a previous work using TiAlSiN-coated tools, in the machining of a hard-to-machine tool-steel. It was found that the wear mechanisms that were present in the coating were adhesion, abrasion, and coating delamination [52]. This was the same coating failure method as registered for both T1 and T2 tool types.

#### 3.4.3. Results Comparison

**Figure 11:**
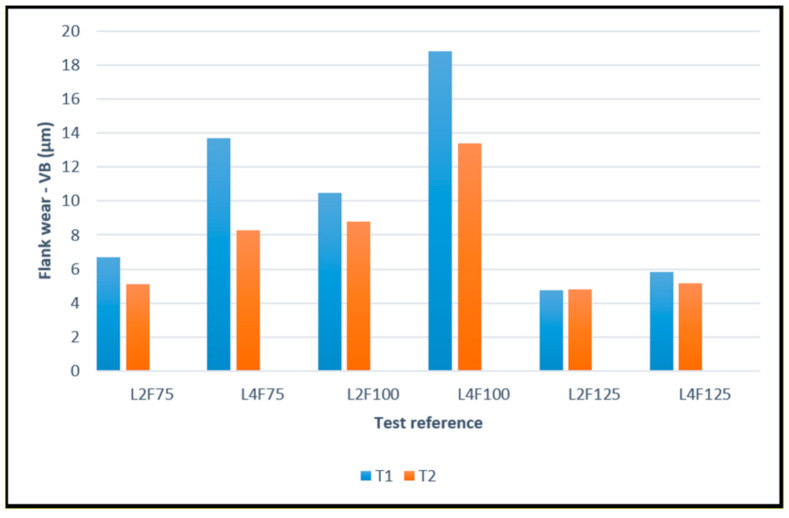
Average values of VB, measured in the clearance faces of T1 and T2 tools, for all the tested cutting conditions.

In this subsection, the average values of VB for both T1 and T2 tools, for all tested conditions, are going to be presented. The identification of each test condition is performed as described in Section 3.3, regarding the comparison of average surface roughness values. The sustained flank wear for each of the tool types can be observed in the following Figure 11.

As seen from Figure 10, both tools exhibit the same wear behavior, with flank wear increasing for higher cutting lengths. For both T1 and T2, a decrease in wear when opting for 75% and 125% feed rate values was registered, with the latter producing the best results in terms of flank wear for both tools. However, the T2 tool type exhibits overall less flank wear, especially for higher cutting lengths, with the wear varying quite little with this parameter. This indicates that the tools coated with HiPIMS suffer less wear, being able to operate longer without suffering considerable flank wear. This can be explained due to the properties that are conferred to the HiPIMS-obtained coatings, which exhibit overall better mechanical properties, when compared to coating’s obtained via more conventional methods [35,36]. These higher values of coating’s mechanical properties promote a better wear behavior on behalf of the coated tool, especially hardness, which can reduce the amount of abrasive wear sustained by the coating [51]. The HiPIMS technique also produces coatings with higher adhesive strength to the substrate [37], which can be related to the wear performance of the coating, with good adhesion to the substrate causing an increase in the coating’s wear resistance [20]. Also worth considering, is the residual stresses present in each coating type, the HiPIMS technique generally produces coatings with higher compressive stresses [38], which can cause an increase in cutting performance and wear behavior [16,51].

## 4. Conclusions

A comparative study of the wear behavior and cutting performance of TiAlSiN coated tools, obtained by two different deposition methods was presented. The following conclusions can be drawn:

- The HiPIMS-obtained coated tools exhibited better results, both in terms of produced machined surface quality and sustained wear, especially for the tests conducted at 4 m cutting length;
- Although better, the TiAlSiN HiPIMS-coated tools produced a very similar machined surface quality to that of the dc MS-obtained coated tools;
- The optimal surface roughness values are obtained for feed rate values of 359.25 mm/min and for cutting length values of 2 m, this is registered for both T1 and T2 tool types;
- Both T1 and T2 tool types presented the same wear behavior, suffering more flank wear for higher cutting lengths and at a feed rate of 479 mm/min (100%);
- There is a significant improvement in the wear behavior of the cutting tool when opting for the HiPIMS-obtained coatings, with these coatings suffering less wear for cutting lengths of 4 m, suffering low variation with cutting parameters;
- Regarding tool wear mechanisms, three predominant mechanisms were identified for both T1 and T2 tool types, these being: adhesion, abrasion and coating delamination;
- A wear cycle consisting of: material adhesion, followed by abrasion in the adhesion area, which in turn promotes coating delamination in the area, which promotes more adhesion in the delaminated coating’s border, was identified for both coated tools;
- Wear mechanism intensity was less severe in the HiPIMS-obtained coated tools, than that registered for the dc MS-obtained tools.

This study highlights the potential of using HiPIMS-obtained coatings with higher mechanical properties in the machining of hard-to-machine alloys, such as the DSS alloys. Although in this case the use of the HiPIMS coating did not produce a significantly better machined surface quality, when compared to the dc MS-obtained coated tool, the increase in wear resistance is quite significant. This can be attributed to the increase in the mechanical properties, obtained from the use of the HiPIMS technique. Furthermore, this technique produces coatings with higher compressive stresses, which is considered beneficial for finishing operations and for the tool’s wear behavior. This coupled with the fact that the HiPIMS technique is highly versatile, makes this novel technique quite appealing, especially when producing coatings that require high mechanical properties and/or increased wear resistance.

## Figures and Tables

**Table 1:** Deposition parameters of the coatings obtained by dcMS and HiPIMS.

Parameters	dc MS	HiPIMS
Deposition time [min.]	240	240
Reactor gases	Ar^+^ + Kr + N_2_	Ar^+^ + Kr + N_2_
Targets’ material	4x TiAlSi 38/57/5	4x TiAlSi 38/57/5
Pressure [mPa]	580	580
Temperature [oC]	450	450
Bias voltage [V]	−110	−110
Target Current Density [A/cm^2^]	20	20
Pulse Frequency [Hz]	-	40
Pulse Duration [µs]	-	400
Holder rotational speed [rpm]	1	1

**Table 2:** Chemical composition (%wt) of the DSS, LDX 2101.

C	Mn	Cu	Cr	Ni	Mo	N
0.03	5.0	0.3	21.5	1.5	0.3	0.22

**Table 3:** Chosen parameters for the machining tests.

Sample	Coating	Deposition Method	Feed Rate (mm/min)	Cutting Length (m)
T1L4F75	TiAlSiN	dc MS	359.25	4
T1L2F75	TiAlSiN	dc MS	359.25	2
T1L4F100	TiAlSiN	dc MS	479	4
T1L2F100	TiAlSiN	dc MS	479	2
T1L4F125	TiAlSiN	dc MS	598.25	4
T1L2F125	TiAlSiN	dc MS	598.25	2
T2L4F75	TiAlSiN	HiPIMS	359.25	4
T2L2F75	TiAlSiN	HiPIMS	359.25	2
T2L4F100	TiAlSiN	HiPIMS	479	4
T2L2F100	TiAlSiN	HiPIMS	479	2
T2L4F125	TiAlSiN	HiPIMS	598.25	4
T2L2F125	TiAlSiN	HiPIMS	598.25	2

**Table 4:** Average hardness, Young’s Modulus, thickness, and H/E ratio values for both TiAlSiN coatings.

Coating	Hardness—H [GPa]	Young’s Modulus—E [GPa]	Thickness (µm)	H/E
TiAlSiN (dcMS)	22.1 ± 0.5	262 ± 9	2.799 ± 0.163	0.084
TiAlSiN (HiPIMS)	31.4 ± 1.2	317 ± 11	2.310 ± 0.122	0.099

**Table 5:** Average surface roughness values, measured in the radial and tangential direction, for tests conducted using T1.

Reference	Radial Ra [µm]	Tangential Ra [µm]
T1L2F75	0.194 ± 0.018	0.175 ± 0.011
T1L4F75	0.253 ± 0.025	0.253 ± 0.018
T1L2F100	0.259 ± 0.016	0.247 ± 0.011
T1L4F100	0.274 ± 0.019	0.284 ± 0.016
T1L2F125	0.250 ± 0.022	0.270 ± 0.014
T1L4F125	0.284 ± 0.033	0.323 ± 0.021

**Table 6:** Average surface roughness values, measured in the radial and tangential direction, for tests conducted using T2.

Reference	Radial Ra [µm]	Tangential Ra [µm]
T2L2F75	0.191 ± 0.013	0.207 ± 0.013
T2L4F75	0.199 ± 0.018	0.224 ± 0.019
T2L2F100	0.206 ± 0.013	0.234 ± 0.009
T2L4F100	0.262 ± 0.021	0.260 ± 0.012
T2L2F125	0.233 ± 0.026	0.227 ± 0.015
T2L4F125	0.286 ± 0.031	0.286 ± 0.023

**Table 7:** Average VB values, obtained from measurements performed in the clearance faces of T1 tools.

Reference	Average VB Values [µm]
T1L2F75	6.710 ± 0.36
T1L4F75	13.71 ± 0.74
T1L2F100	10.47 ± 0.66
T1L4F100	18.80 ± 1.16
T1L2F125	4.770 ± 0.22
T1L4F125	5.830 ± 0.65

**Table 8:** Average VB values, obtained from measurements performed in the clearance faces of T2 tools.

Reference	Average VB Values [µm]
T2L2F75	5.13 ± 0.09
T2L4F75	8.26 ± 0.14
T2L2F100	8.8 ± 0.11
T2L4F100	13.41 ± 1.19
T2L2F125	4.82 ± 0.24
T2L4F125	5.14 ± 0.12

## References

[1] Cheng X., Wang Y., Li X., Dong C. (2018). Interaction between austenite-ferrite phases on passive performance of 2205 duplex stainless steel. J. Mater. Sci. Technol..

[2] Vinoth Jebaraj A., Ajaykumar L., Deepak C.R., Aditya K.V.V. (2017). Weldability, machinability and surfacing of commercial duplex stainless steel AISI2205 for marine applications—A recent review. J. Adv. Res..

[3] Nomani J., Pramanik A., Hilditch T., Littlefair G. (2015). Chip formation mechanism and machinability of wrought duplex stainless steel alloys. Int. J. Adv. Technol..

[4] Chail G., Kangas P. (2016). Super and hyper duplex stainless steels: Structures, properties, and applications. Procedia Struct. Integrity.

[5] Nomani J., Pramanik A., Hilditch T., Littlefair G. (2013). Machinability study of first generation duplex (2205), second generation duplex (2507) and austenite stainless steel during drilling process. Wear.

[6] Koyee R.D., Heisel U., Eisseler R., Schmauder S. (2014). Modeling and optimization of turning duplex stainless steels. J. Manuf. Processes.

[7] Gowthaman P.S., Jeyakumar S., Saravanan B.A. (2020). Machinability and tool wear mechanism of Duplex stainless steel—A review. Mater. Today Proc..

[8] Sahithi V.V.D., Malayadrib T., Srilatha N. (2019). Optimization of Turning Parameters on Surface Roughness Based on Taguchi Technique. Mater. Today Proc..

[9] Tlhabadira I., Daniyan I.A., Masu L., Van Staden L.R. (2019). Process Design and Optimization of Surface Roughness during M200 TS Milling Process using the Taguchi Method. Procedia CIRP.

[10] Vishnu Vardhan M., Sankaraiah G., Yohan M., Jeevan Rao H. (2017). Optimization of Parameters in CNC milling of P20 steel using Response Surface methodology and Taguchi Method. Mater. Today Proc..

[11] Zhang J.Z., Chen J.C., Kirby E.D. (2007). Surface roughness optimization in an end-milling operation using the Taguchi design method. J. Mater. Process. Technol..

[12] Selvaraj D.P. (2017). Optimization of cutting force of duplex stainless steel in dry milling operation. Mater. Today Proc..

[13] Airao J., Chaudhary B., Bajpai V., Khanna N. (2018). An Experimental Study of Surface Roughness Variation in End Milling of Super Duplex 2507 Stainless Steel. Mater. Today Proc..

[14] Policena M.R., Devitte C., Fronza G., Garcia R.F., Souza A.J. (2018). Surface roughness analysis in finishing end-milling of duplex stainless steel UNS S32205. Int. J. Adv. Manuf. Technol..

[15] Sousa V.F.C., Silva F.J.G. (2020). Recent Advances in Turning Processes Using Coated Tools—A Comprehensive Review. Metals.

[16] Sousa V.F.C., Silva F.J.G. (2020). Recent Advances on Coated Milling Tool Technology—A Comprehensive Review. Coatings.

[17] Martinho R.P., Silva F.J.G., Baptista A.P.M. (2008). Cutting forces and wear analysis of Si3N4 diamond coated tools in high speed machining. Vacuum.

[18] Paiva J.M.F., Amorim F.L., Soares P.C., Veldhuis S.C., Mendes L.A., Torres R.D. (2019). Tribological behavior of superduplex stainless steels against PVD hard coatings on cemented carbide. Int. J. Adv. Manuf. Technol..

[19] Silva F.J.G., Casais R.C.B., Martinho R.P., Baptista A.P.M. (2012). Mechanical and Tribological Characterization of TiB_2_ Thin Films. J. Nanosci. Nanotechnol..

[20] Silva F.J.G., Martinho R.P., Alexandre R.J.D., Baptista A.P.M. (2012). Wear Resistance of TiAlSiN Thin Coatings. J. Nanosci. Nanotechnol..

[21] Silva F.J.G., Martinho R., Andrade M., Baptista A.P.M., Alexandre R. (2017). Improving the Wear Resistance of Moulds for the Injection of Glass Fibre-Reinforced Plastics Using PVD Coatings: A Comparative Study. Coatings.

[22] Silva F.J.G., Martinho R.P., Alexandre R.J.D., Baptista A.P.M. (2011). Increasing the wear resistance of molds for injection of glass fiber reinforced plastics. Wear.

[23] Nunes V., Silva F.J.G., Andrade M.F., Alexandre R., Baptista A.P.M. (2017). Increasing the lifespan of high-pressure die cast molds subjected to severe wear. Surf. Coat. Technol..

[24] Silva F.J.G., Fernandes A.J.S., Costa F.M., Teixeira V., Baptista A.P.M., Pereira E. (2003). Tribological behaviour of CVD diamond films on steel substrates. Wear.

[25] Silva F.J.G., Baptista A.P.M., Pereira E., Teixeira V., Fan Q.H., Fernandes A.J.S., Costa F.M. (2002). Microwave plasma chemical vapour deposition diamond nucleation on ferrous substrates with Ti and Cr Interlayers. Diamond Relat. Mater..

[26] Silva F.J.G., Fernandes A.J.S., Costa F.M., Baptista A.P.M., Pereira E. (2004). A new interlayer approach for CVD diamond coating of steel substrates. Diamond Relat. Mater..

[27] Baptista A., Silva F.J.G., Porteiro J., Míguez J.L., Pinto G. (2018). Sputtering physical vapour deposition (PVD) coatings: A critical review on process improvement and market trend demands. Coatings.

[28] Ginting A., Skein R., Cuaca D., Herdianto P., Masyithah Z. (2018). The characteristics of CVD- and PVD-coated carbide tools in hard turning of AISI 4340. Measurement.

[29] Koseki S., Inoue K., Morito S., Ohba T., Usuki H. (2015). Comparison of TiN-coated tools using CVD and PVD processes during continuous cutting of Ni-based superalloys. Surf. Coat. Technol..

[30] Caliskan H., Panjan P., Kurbanoglu C. (2017). Hard coatings on cutting tools and surface finish. Compr. Mater. Finish..

[31] Paiva J.M.F., Torres R.D., Amorim F.L., Covelli D., Tauhiduzzaman M., Veldhuis S., Dosbaeva G., Fox-Rabinovich G. (2017). Frictional and wear performance of hard coatings during machining of superduplex stainless steel. Int. J. Adv. Manuf. Technol..

[32] Klocke F., Krieg T. (1999). Coated tools for metal cutting—Features and applications. CIRP Ann..

[33] Fernández-Abia A.I., Barreiro J., Fernández-Larrinoa J., de Lacalle L.N.L., Fernández-Valdivielso A., Pereira O.M. (2013). Behaviour of PVD Coatings in the Turning of Austenitic Stainless Steels. Procedia Eng..

[34] Baptista A., Silva F.J.G., Porteiro J., Míguez J.L., Pinto G., Fernandes L. (2018). On the Physical Vapour Deposition (PVD): Evolution of Magnetron Sputtering Processes for Industrial Applications. Procedia Manuf..

[35] Ma Q., Li L., Xu Y., Gu J., Wang L., Xu Y. (2017). Effect of bias voltage on TiAlSiN nanocomposite coatings deposited by HiPIMS. Appl. Surf. Sci..

[36] Zhao B., Zhao X., Lin L., Zou L. (2020). Effect of bias voltage on mechanical properties, milling performance and thermal crack propagation of cathodic arc ion-plated TiAlN coatings. Thin Solid Films.

[37] Zauner L., Ertelthaler P., Wojcik T., Bolvardi H., Kolozsvári S., Mayrhofer P.H., Riedl H. (2020). Reactive HiPIMS deposition of Ti-Al-N: Influence of the deposition parameters on the cubic to hexagonal phase transition. Surf. Coat. Technol..

[38] Tillmann W., Grisales D., Stangier D., Thomann C.-A., Debus J., Nienhaus A., Apel D. (2020). Residual stresses and tribomechanical behaviour of TiAlN and TiAlCN monolayer and multilayer coatings by DCMS and HiPIMS. Surf. Coat. Technol..

[39] Vasu M., Nayaka H.S. (2018). Investigation of Cutting Force Tool Tip Temperature and Surface Roughness during Dry Machining of Spring Steel. Mater. Today Proc..

[40] Phokobye S.N., Daniyan I.A., Tlhabadira I., Masu L., VanStaden L.R. (2019). Model Design and Optimization of Carbide Milling Cutter for Milling Operation of M200 Tool Steel. Procedia CIRP.

[41] Strafford K.N., Audy J. (1997). Indirect monitoring of machinability in carbon steels by measurement of cutting forces. J. Mater. Process. Technol..

[42] Venkatesan K., Manivannan K., Devendiran S., Mathew A.T., Ghazaly N.M., Aadhavan, Benny S.M.N. (2019). Study of Forces, Surface Finish and Chip Morphology on Machining of Inconel 825. Procedia Manuf..

[43] Sousa V.F.C., Silva F.J.G., Fecheira J.S., Lopes H.M., Martinho R.P., Casais R.B., Ferreira L.P. (2020). Cutting Forces Assessment in CNC Machining Processes: A Critical Review. Sensors.

[44] Gouveia R., Reis P., Baptista A. (2016). Machining duplex stainless steel: Comparative study regarding end mill coated tools. Coatings.

[45] Seid Ahmed Y., Paiva J., Covelli D., Veldhuis S. (2017). Investigation of Coated Cutting Tool Performance during Machining of Super Duplex Stainless Steels through 3D Wear Evaluations. Coatings.

[46] Dos Santos A.G., da Silva M.B., Jackson M.J. (2018). Tungsten carbide micro-tool wear when micro milling UNS S32205 duplex stainless steel. Wear.

[47] Diniz A.E., Machado A.R., Corrêa J.G. (2016). Tool wear mechanisms in the machining of steels and stainless steels. Int. J. Adv. Manuf. Technol..

[48] Krolczyk G.M., Nieslony P., Legutko S. (2015). Determination of tool life and research wear during duplex stainless steel turning. Arch. Civ. Mech. Eng..

[49] Rajaguru J., Arunachalam N. (2017). Coated tool Performance in Dry Turning of Super Duplex Stainless Steel. Procedia Manuf..

[50] Suárez A., López de Lacalle L.N., Polvorosa R., Veiga F., Wretland A. (2017). Effects of high-pressure cooling on the wear patterns on turning inserts used on alloy IN718. Mater. Manuf. Process..

[51] Sousa V.F.C., Silva F.J.G., Pinto G.F., Baptista A., Alexandre R. (2021). Characteristics and Wear Mechanisms of TiAlN-Based Coatings for Machining Applications: A Comprehensive Review. Metals.

[52] Sousa V.F.C., Silva F.J.G., Alexandre R., Fecheira J.S., Silva F.P.N. (2021). Study of the wear behaviour of TiAlSiN and TiAlN PVD coated tools on milling operations of pre-hardened tool steel. Wear.

[53] Ghailane A., Makha M., Lharlimi H., Alami J. (2020). Design of hard coatings deposited by HiPIMS and dcMS. Mater. Lett..

[54] (1986). ISO 8688-2:1986—Tool Life Testing in Milling—Part 2: End Milling.

